# Influence of a passive shoulder exoskeleton on drilling performance in women- a cross-sectional study

**DOI:** 10.1038/s41598-026-53198-9

**Published:** 2026-06-09

**Authors:** Julia Katharina Gräf, Bettina Wollesen, María Alejandra Díaz, Sander De Bock, Lasse Hansen, Vincent Ducastel, Alessandra Preckher, Bart Roelands, Kevin De Pauw

**Affiliations:** 1 Institute of Human Movement Science, University of Hamburg, Hamburg, Germany; 2 Institute of Movement Therapy and Movement-oriented Prevention and Rehabilitation, German Sports University Cologne, Cologne, Germany; 3 Human Physiology and Sports Physiotherapy research group (MFYS), Vrije Universiteit Brussel (VUB), Ixelles, Belgium; 4 Brussels Human Robotics Research Center (BruBotics), Brussels, Belgium; 5 Institute of Biomechanics and Orthopaedics, German Sport University Cologne, Cologne, Germany; 6 Robotics & MultiBody Mechanics Research Group, Vrije Universiteit Brussel (VUB), Ixelles, Belgium; 7 IMEC Brussels, Brussels, Belgium; 8 Axiles Bionics, Brussels, Belgium

**Keywords:** Upper-body exoskeleton, Muscle synergy, Overhead work, User comfort, Engineering, Health care

## Abstract

Work-related musculoskeletal disorders are common, especially among women performing repetitive overhead tasks. In a randomized 2 × 2 crossover study with 14 female participants, we investigated the effects of a passive upper-body exoskeleton during an overhead precision task, involving the tightening of 20 bolts into a sensor-based workstation, while muscle activation, task performance, and usability were assessed. The results showed significant reduced M. Trapezius muscle activations during arm lowering (p = .041, 73%), and lower target accuracy (p < .001, 100%) when using the exoskeleton. Subjective strain in the shoulders was significantly lower when using the exoskeleton (p = .035, 15%). The usability was rated as “unacceptable”, with users criticizing the complexity and learning effort. While the exoskeleton reduced muscle load, its mechanical limitations impaired precision and usability, especially for women. These results highlight the importance of sex-specific, ergonomic, and adaptive designs to improve exoskeleton effectiveness and acceptance.

## Introduction

Work-related musculoskeletal disorders (WMSDs) are a significant occupational health concern, particularly for tasks requiring repetitive arm and hand movements, such as overhead work^1^. According to the European Occupational Health and Safety Report (2019), 58% of employees suffer from WMSDs. The main WMSDs are neck, shoulder, and back pain, resulting in poorer health-related well-being, affecting people worldwide^2^; World Health Organization, 2022. Women have a higher prevalence (22%- 47%) of WMSDs compared to men^3–6^(odds ratio 1.5), and prevalence also increases with age with a peak between 65 and 69 years European Agency for Safety and Health at Work, 2019^3^. A specific risk factor for WMSDs is the repetitive lifting of loads in non-ergonomic postures, especially in overhead positions^1^. Overhead tasks are highly relevant in different branches, such as manual occupations^7^ however, this overhead work also increases the risk of shoulder and neck pain by 48%. Barthelme et al.^7^,. To address these demands, occupational exoskeletons have emerged as a promising intervention, aiding the arms, shoulders, and torso^8^. They can provide mechanical support during activities such as lifting, reaching, and carrying objects, reduce physical strain, promote ergonomic posture and movement, and reduce the level of fatigue^9^r et al., 2021^10–13^ Gillette and Stephenson^14–17^.

Reviews have explored passive exoskeleton effects on task performance and worker perception. Fournier^18^, examined the influence of exoskeleton use on quality and productivity measures (e.g., endurance time, task completion time, number of errors, and number of task cycles completed) and revealed mixed results about the impact of exoskeleton use, dependent on task characteristics, e.g. reduced completion task time during a repetitive overhead drilling task by 20%^19^ or static holding endurance time from 3.2 min up to 9.7 min^20^. However, it must be noted that within the integrated 15 studies, only five studies examined female participants, and the authors did not provide any information on whether the performance differed between males and females. Similarly, Kuber et al.^8^, Ashta et al.^21^, and Brambilla et al.^22^, indicated that the usage of upper-limb exoskeletons may influence the cognitive workload and physical performance, with outcomes ranging from positive to negative depending on the task and the specific design of the device. For instance, using an exoskeleton may demand additional focus to manage the device, adjust movements, and maintain balance^23^, creating an environment that requires a high degree of multitasking^24^. For example, during overhead activities performed with a passive exoskeleton, a 34% reduction in muscle activation of the deltoid muscle and a 15–21% reduction in the trapezius muscle were observed^25,26^.

Unfortunately, the existing studies on industrial exoskeletons include an 80% male cohort^11^, highlighting the lack of knowledge on the impact of the use of exoskeletons on women. Recent efforts addressed this gap, for example, Tyagi et al.^27^, observed that female participants experienced particularly pronounced reductions in shoulder muscle activity compared to male participants. Moreover, Wollesen et al., 2024a^28^ emphasized the importance of examining sex (Sex generally refers to a set of biological attributes that are associated with physical and physiological features such as chromosomal genotype, hormonal levels, internal and external anatomy. A binary sex categorization (male/female) is usually designated at birth ("sex assigned at birth") and is in most cases based solely on the visible external anatomy of a newborn. In reality, sex categorizations include people who are intersex/have differences of sex development (DSD)) differences when using occupational exoskeletons, such as higher values in male participants for task performance (error integrals, *p* <.001); however, the authors also provided some results that body composition might have more impact on working performance than sex^28^.

Beyond muscle activation, it is essential to examine how exoskeletons influence muscle synergy patterns. Several studies have already shown that the central nervous system can simplify complex movements by grouping co-activated muscles into modular organizational units, called muscle synergies (Bernstein, 1967; Flash and Hochner, 2005; Tresch et al., 2002; Krishnamoorthy et al., 2007). Muscle synergies indicate the relative activation levels of muscles to a synergy, where the absolute activation level is modulated by a single neural command^29^. This means that while each muscle in the synergy maintains a fixed contribution ratio, the overall intensity of activation can increase or decrease depending on the strength of the neural input. So far, the effects of an industrial (upper body) exoskeleton on muscle synergies have only been investigated by few studies (Cohic et al., 2025; Park Nussbaum, 2025, Tian et al., 2025)^30^. Passive exoskeletons may unexpectedly affect these coordination between agonist and antagonist muscles, potentially leading to unintended co-contraction or suppressed activation (Mussa-Ivaldi et al., 1994^31^. Thus, closely monitoring muscle activation patterns and muscle synergies is essential to better understand and optimize exoskeleton use for ergonomic benefits by identifying imbalances such as overuse, suppressed activation, or increased co-contraction, and adjusting assistive force, alignment, or stiffness to support natural coordination and reduce fatigue. However, understanding these effects could be crucial in analysing the complex interaction between the user and the exoskeleton.

Furthermore, an inappropriate exoskeleton fit can lead to discomfort and possibly alter body kinematics and increase the risk of injury. McFarland et al.^32^, point out that the extent of these changes is unclear. Moreover, studies show sex-specific differences; while Leibman and Choi^33^ observed no differences between sex for the upper-limb exoskeleton fit and pain during working tasks, Gutierrez et al.^34^, noted that women had more barriers to exoskeleton use, assuming that the fitting of an exoskeleton will not capture the anthropometric differences and therefore using an exoskeleton might induce discomfort.

Taken together, these findings point out three critical gaps. First, most exoskeleton research has focused on male populations, limiting generalizability to women, who generally have lower muscular strength and different anthropometry compared to men, which increases the risk of MSDs. Sex differences should therefore be taken into consideration, especially when developing and adapting exoskeletons for female users. Second, although reduced muscle activation is well documented, there is a lack of comprehensive analyses of how exoskeletons reshape muscle coordination patterns, as captured by synergy models. Third, performance metrics (e.g., accuracy, speed) and subjective experiences (comfort, usability) have not been systematically compared in female-only cohorts.

The present study addresses these gaps by evaluating the effects of an upper-body exoskeleton (Exo4Work) during overhead work in a female cohort. Specifically, we investigate changes in shoulder and arm muscle activity and synergy patterns with and without exoskeleton assistance. Additionally, we investigate task performance, measured in terms of accuracy and duration, and female participants’ perceptions of the exoskeleton comfort and technical experience.

We hypothesized that the exoskeleton would reduce shoulder and arm muscle activity compared to unassisted condition. Given that female participants are underrepresented in exoskeleton research and typically exhibit lower absolute upper-body strength and different anthropometric characteristics, the magnitude of relative muscle unloading may differ from vales reported in predominantly male samples. Therefore, potentially larger reductions in muscle activity may be observed in this female cohort compared to previous findings.

Consistent with previous findings that external support can alter neuromuscular control strategies, changes in muscle coordination patterns may occur when using an exoskeleton. However, these changes are considered provisional and may manifest more as alterations in co-activation patterns than as a systematic increase in antagonist activity.

Accordingly, we also anticipated possible changes in task performance (accuracy and duration) as well as generally positive subjective impressions regarding wearing comfort during the drilling exercise.

## Materials and methods

### Compliance with ethical standards

This study was conducted in accordance with the standards of the Declaration of Helsinki and the local ethical commission (Vrije Universiteit Brussel and Universitair Ziekenhuis Brussel, B.U.N.: 143201941463). All participants provided written informed consent before the study.

### Study design

This study followed a within-subject randomized crossover study design. The participants were randomly assigned to perform the task with and without the exoskeleton, ensuring balanced exposure to each condition. To complete the study protocol, participants attended the laboratory on two separate days, allowing for adequate assessment under exoskeleton and non-exoskeleton conditions.

### Participants

#### Sample size calculation

A power analysis using GPower (matched pairs, dz = 0.56, alpha = 0.05, power = 0.8) indicated a required sample size of 22. Accordingly, 21 female participants without prior exoskeleton or industrial task experience could be recruited at or near the VUB campus. Due to poor EMG data (disturbed EMG signals), 7 participants were excluded, resulting in a final sample of 14 women (27 ± 10 years; 163.5 cm ± 3.7 cm; 60.7 kg ± 9.1 kg) for analysis.

### Measurements

#### Surface electromyography (sEMG)

sEMG sensors were placed on the right side of the body, specifically targeting the following muscles: Trapezius (tr), all three heads of the Deltoideus (anterior da, medialis dm, posterior dp), Brachioradialis (br), Biceps brachii (bb), and Triceps brachii (tlh). Before locating the sensors, skin preparation was conducted according to SENIAM guidelines (Hermens et al., 1999; Barbero et al., 2011). To quantify muscle activity, three standardized maximal voluntary isometric contractions (MVC) over 7 s were performed for each monitored muscle. The MVC value was calculated as the average peak activity from the two highest-contraction trials. EMG data were collected at a sampling rate of 2000 Hz using the Cometa MiniWave system (Italy).

#### Passive shoulder exoskeleton characteristics

In this study, the Exo4Work passive shoulder exoskeleton was used. This exoskeleton is described in de Bock et al.^10,11^, and is part of ongoing research and development collaboration. Previous research^10,11,35^ has already demonstrated its positive impacts on various parameters during above-shoulder work. However, this exoskeleton was not designed specifically for women. Although the level of assistance provided by the exoskeleton can be adjusted by changing the pretension of the spring, the exoskeleton provides estimated peak assistance of 3 Nm^11^. The exoskeleton is worn like a backpack and features a hip belt, shoulder straps, and a chest belt for secure positioning. The upper arms were secured using Velcro straps, similar to commercially available devices. The exoskeleton was individually adjusted for wearer comfort and adds 3.8 kg of weight to the body.

#### RPE and body part discomfort

The subjective assessment of the perceived effort was carried out after each trial using the 100-point Borg scale (0–100). 0 corresponds to a very, very low level of effort, while 100 means a very, very high level of effort^36^. The rating refers to the overall perceived exertion. Furthermore, for the body-part discomfort scale (BPDS) for specific body regions such as head and neck, shoulders, arms, upper and lower back, buttocks, thighs, knees, lower legs, and feet, chest region, abdominal region, and front of pelvis, was used with a discomfort level of 1 indicating comfortable to 5, extremely uncomfortable (Corlett & Bishop, 1976).

#### System usability scale

The System Usability Scale (SUS) is a reliable instrument for measuring user interaction. It consists of ten questions with possible answers ranging from ‘strongly agree’ to ‘strongly disagree’. It provides an overall measure of usability, referring to how easily a user can accomplish their goals when using this device, including user satisfaction and success rates^37,38^.

### Procedures

Upon initial arrival, the subject was informed about the protocol and signed an informed consent. All tests took place at the laboratory of the Human Physiology and Sports Physiotherapy Research Group (MFYS, VUB). The experiment consisted of a total of three laboratory visits in which general participant characteristics (e.g., body height and weight, measurements of body parts) were initially recorded (visit 1) and participants were introduced to the experimental protocol, the laboratory environment, and the Exo4Work exoskeleton (familiarization of around 1 h). Over the following two visits, the participants completed the experimental protocol. Each trial lasted approximately 1.5 h. In between the first and the second visit, at least 48 h were scheduled. In between the second and the third laboratory visit, 6 to 9 days were foreseen.

#### Familiarization

The first visit to the lab included familiarization with the study protocol and the exoskeleton to get to know the routine and to reduce learning effects throughout the experimental trials. This involved the execution of the overhead precision task with the Exo4Work exoskeleton^39^ up to 12 times with a 3-minute break in between each trial. The overall familiarization process lasted approximately about 1.5 h to accustom participants to the experimental tasks, measurement devices and the use of the exoskeleton.

#### Experimental protocol

The second and third visit involved the experimental trials, which included the execution of a custom precision task under randomized exoskeleton and non-exoskeleton conditions, following the protocol by De Bock et al.^11^, and based on methods developed by Kim et al.^19^, to evaluate overhead work precision. No further familiarization was carried out.

After locating the sEMG sensors and performing the 3 MVC tests, the Exo4Work was applied and adjusted to the participant with the assistance of the investigators. After each trial, the RPE and a local BPDS were filled out. Additionally, the SUS was filled out at the end of the session with the exoskeleton.

#### Overhead precision task

**Fig. 1:**
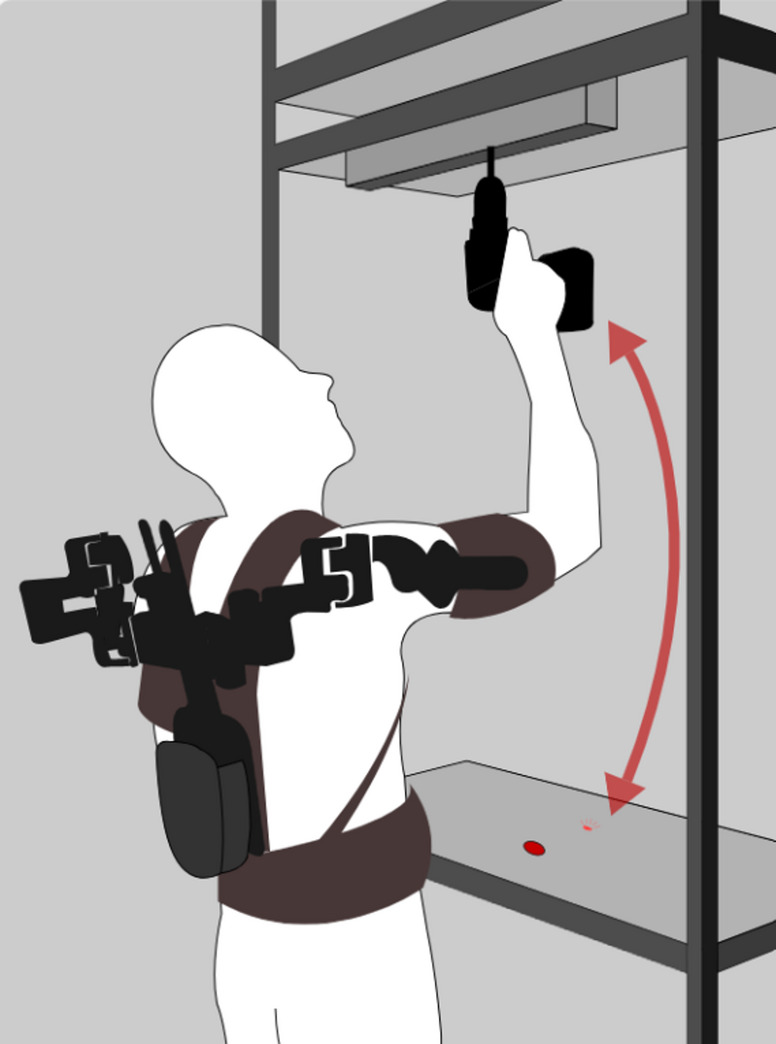
Drilling task performance.

Participants utilized a Black & Decker electric screwdriver (1.14 kg) to tighten 20 bolts pre-inserted into an aluminium plate positioned overhead (cf. Figure 1). Force sensors and accelerometers were integrated into the overhead working setup to quantify working performance (duration and accuracy) and to facilitate the segmentation of acquired signals^11^ Wollesen et al., 2024). Participants pressed a push button at pelvic crest height, tightened a bolt at overhead height, and pressed the button again to indicate movement initiation (as indicated in Fig. 1). Sensorized aluminium and plexiglass plates allowed tracking contact between the screwdriver bit and the bolt, as well as monitoring screwing errors. The appropriate overhead height was determined using the method described by Sood et al.^40^, which calculates hand height with the shoulder and elbow at a 90-degree angle, plus 0.4 times the difference between hand height with the arm fully extended and hand height at the 90-degree angle.

Figure 1 shows the overhead precision task setup: participants used a 1.14 kg electric screwdriver to tighten 20 bolts in an overhead aluminium plate, with sensors tracking performance, movement, and errors.

### Analysis

#### EMG

For consistency, the second trial was used, as the first and last trials often exhibited an increased susceptibility to poor-quality data such as excessive noise, motion artifacts, low signal-to-noise ratio or absent muscle activation patterns, likely due to initial sensor setup and subsequent electrode displacement. The raw EMG signals were bandpass filtered (Butterworth, 4th order, 20–500 Hz), rectified, and smoothed using a 100ms root mean square (RMS). Subsequently, the EMG signal quality was visually inspected, and 7 participants were excluded due to excessive noise or artifacts that prevented reliable EMG interpretation. This resulted in a final sample size of 14 participants. The exclusions were not condition-specific and occurred in both experimental conditions, which reduced the likelihood of systematic bias between conditions. However, the relatively high exclusion rate may have reduced statistical power and should be taken into account when interpreting the results. Signals were then normalized to the MVC values. The 20 repetitions were cut into 20 separate cycles (from lifting the arm, drilling, lowering the arm), excluding irrelevant movements, and averaged per participant using a 200-point interpolation. The complete movement cycle (lifting, drilling, and lowering) was analyzed as a single continuous cycle.

#### Muscle synergies

To identify the muscle synergies, a non-negative matrix factorization was performed for each participant using three synergies with an imposed factorization rank of three, determined based on the variance accounted for (VAF) criterion with 50 runs (global VAF = 90%, local VAF = 85%). The EMG data were structured as a muscle-by-time matrix with normalized time points across movement cycles. The NNMF analysis was performed using random initialization, default convergence criteria, and a maximum of 100 iterations. This analysis results in weighted muscle vectors (W) that define the relative contribution of each muscle to a synergy, while H refers to the activation coefficients that capture the time-varying activation level of each synergy across tasks or conditions. Finally, muscle synergies were clustered based on their activation patterns (H) into three distinct clusters via k-means clustering (MATLAB k-means + + algorithm)^41^. Multiple iterations were used, and the final clustering solution was selected based on the lowest sum of squares within the clusters. The peak position and full width at half maximum (FWHM) were calculated post hoc from the cluster means derived from H to characterize temporal activation features. The peak position was defined as the time of maximum activation within the normalized cycle, while the FWHM quantified the duration of activation. Reported will also be W, representing the clustering towards the minimal variance in H. The resulting muscle weights were then used for statistical calculations, and the activation patterns were used to describe the movement phases of arm elevation up to the drilling point - drilling phase where the drilling takes place - and arm elevation phase where the arm is removed to the neutral position. The data was organized into three clusters, each corresponding to a different phase of the movement.

#### Performance

Performance data, including task, aiming, and drilling duration as well as error integral during aiming and error integral during drilling, were analysed and reported as mean ± standard deviation (SD), with statistical comparisons conducted using repeated measures ANOVA to assess within-subject differences between *Exo* and *noExo* conditions.

#### RPE and SUS

The Borg Scale RPE and BPDS data were analyzed and reported as mean ± SD, as well as 95% CI and effect sizes to summarize central tendency and variability for overall, (cf. 2.3).

SUS scores were calculated according to Brooke^38^and Bangor et al.^37^, yielding values ranging from 0 to 100, with 100 representing the best imaginable and highly acceptable usability. Scores below 50 indicate unacceptable usability, and scores between 50 and 70 are considered marginally acceptable.

#### Statistical procedures

For all statistical analyses, normality of distribution was tested using Kolmogorov-Smirnov. The corresponding comparative test was then selected. The Wilcoxon-signed rank test was employed for the muscle synergy data using W, as the data is clustered towards minimal variance in H, the RPE and BPDS data, while repeated measures ANOVA (with *Exo* and *noExo* as within-subject factors) was utilized for the performance data. Where applicable, corrections for multiple comparisons were applied using the Bonferroni method. All analyses were performed using MATLAB 2019. a, Python 3.6, Microsoft Excel 2016, and SPSS 29.0, with alpha set at 0.05.

## Results

### Muscle synergy

**Fig. 2:**
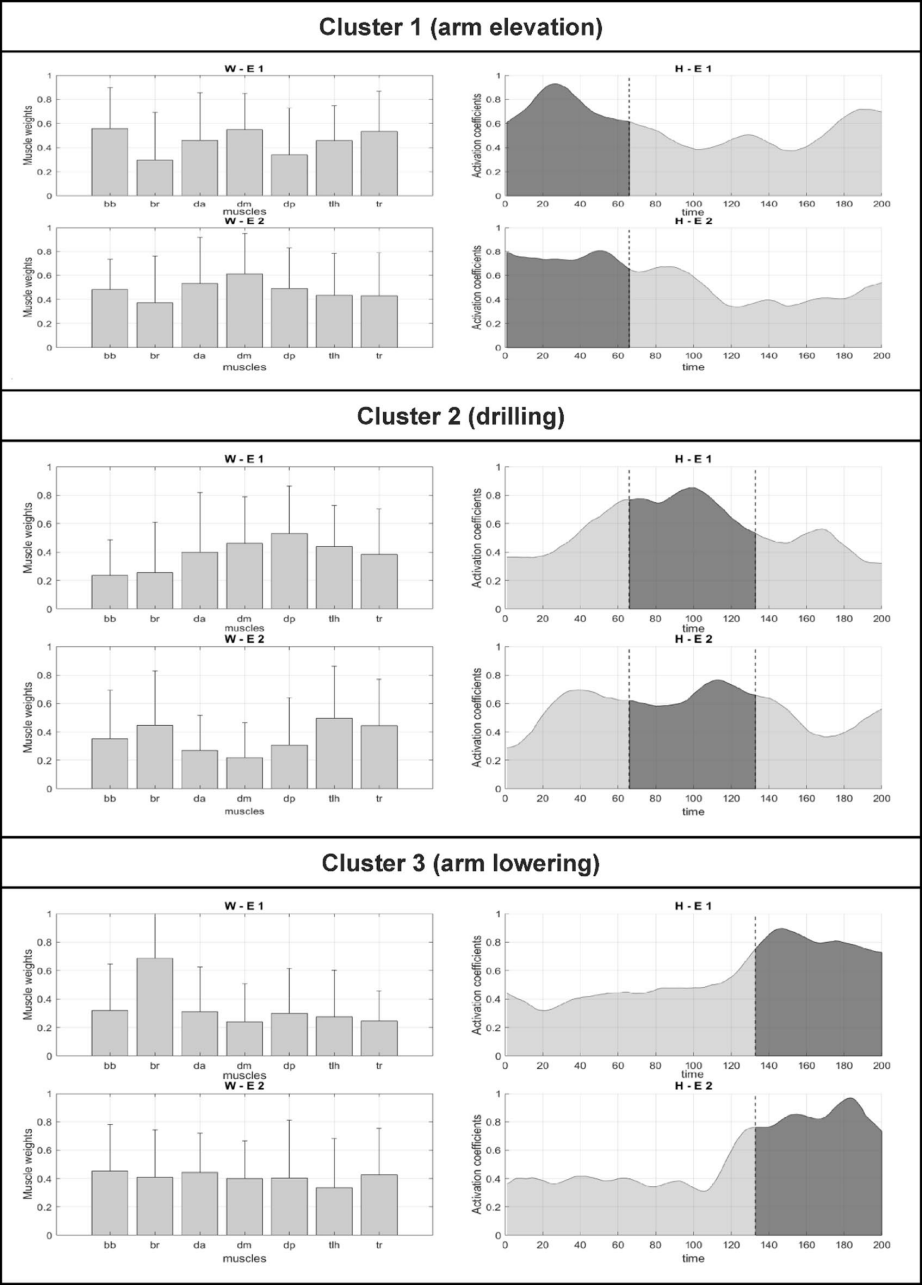
Muscle synergy cluster analysis. E1 = noExo, E2 = Exo.; W = the time-invariant muscle synergy vectors; H=time-dependent activation coefficients; Anterior Deltoid (da), Medial Deltoid (dm), Posterior Deltoid (dp), Biceps Brachii (bb), Long Head of Triceps Brachii (tlh), Brachioradialis (br), Trapezius Descendens (tr).

Figure 2 shows muscle synergy cluster analysis with and without exoskeleton across three task phases: Cluster 1 (arm elevation), Cluster 2 (drilling), and Cluster 3 (arm lowering). Each cluster presents bar charts of weighted muscle activations for seven muscles and line plots of corresponding activation patterns. Without the exoskeleton, some muscles show higher activation in specific phases, while with the exoskeleton, activations appear more evenly distributed across muscles.

#### No Exo (E1)

In Cluster 1 (arm elevation), the muscles *tr* and *da* showed relatively higher mean weights compared to the other muscles, indicating their prominent roles during arm elevation. The other muscles exhibit lower activation levels, though they still contribute to the movement.

In Cluster 2 (drilling phase), muscle weights across all muscles appeared more balanced compared to Cluster 1, suggesting that stabilization is well distributed.

In Cluster 3 (arm returning), the muscle weights of all seven muscles remained relatively consistent, except for the br with higher activation compared to the other involved muscles.

Overall, the muscles *tlh* and *dm* consistently showed higher levels of activation across clusters compared to other muscles (cf. Table 1).

#### Exo (E2)

In Cluster 1 (arm elevation phase), the muscle weights were relatively consistent across all muscles and contributed moderately, indicating a well-distributed muscle engagement.

Similar to Cluster 1, the mean weights across all muscles in Cluster 2 (drilling phase) remained evenly distributed, suggesting that the stabilization and effort required during the drilling task were shared among all muscles.

In Cluster 3 (arm returning phase), the weighted muscles were also balanced, similar to Clusters 1 and 2, with consistent and controlled muscle engagement.

Furthermore, the comparison of the muscles between the *Exo* and *noExo* conditions revealed that the muscle weights slightly differ between both conditions. A significant difference was observed for the *tr* during Cluster 3 (arm returning phase), with higher activation in the *Exo* condition compared to *noExo* condition (*Z* = −2.040 and *p* =.041) (cf. Table 1). For other muscles and clusters, although the trends in muscle weights differed qualitatively between both conditions, no statistically significant differences were found (cf. Table 1).

**Table 1:** Weighted muscle data.

Muscle	noExo [M ± SD] (95% CI)	Exo [M ± SD] (95% CI)	Z	*p*	Effect size (rank-biserial correlation)
Cluster 1					
bb	0.559 ± 0.338 (0.363–0.754)	0.484 ± 0.253 (0.338–0.630)	−0.534	0.594	0.200
br	0.298 ± 0.394 (0.070–0.525)	0.376 ± 0.387 (0.152–0.599)	−0.549	0.583	0.333
da	0.458 ± 0.398 (0.228–0.688)	0.534 ± 0.383 (0.313–0.755)	−0.345	0.730	−0.200
dm	0.548 ± 0.301 (0.374–0.722)	0.614 ± 0.336 (0.420–0.808)	−0.471	0.638	−0.067
dp	0.338 ± 0.387 (0.115–0.561)	0.492 ± 0.340 (0.296–0.688)	−1.083	0.279	−0.275
tlh	0.456 ± 0.292 (0.287–0.624)	0.435 ± 0.348 (0.234–0.636)	−0.031	0.975	−0.276
tr	0.532 ± 0.336 (0.338–0.726)	0.432 ± 0.355 (0.227–0.637)	−1.223	0.221	−0.067
Cluster 2					
bb	0.236 ± 0.250 (0.092–0.250)	0.353 ± 0.341 (0.157–0.550)	−0.734	0.463	−0.890
br	0.257 ± 0.351 (0.054–0.460)	0.447 ± 0.393 (0.226–0.668)	−1.293	0.196	−0.795
da	0.399 ± 0.421 (0.156–0.642)	0.270 ± 0.249 (0.126–0.413)	−0.454	0.650	0.165
dm	0.461 ± 0.330 (0.271–0.652)	0.217 ± 0.244 (0.076–0.358)	−1.852	0.064	0.543
dp	0.530 ± 0.336 (0.336–0.724)	0.306 ± 0.336 (0.112–0.500)	−1.852	0.064	0.410
tlh	0.438 ± 0.290 (0.271–0.606)	0.496 ± 0.365 (0.285–0.707)	−0.596	0.511	−0.105
tr	0.384 ± 0.319 (0.199–0.568)	0.443 ± 0.327 (0.255–0.632)	−0.785	0.433	−0.162
Cluster 3					
bb	0.320 ± 0.325 (0.132–0.508)	0.454 ± 0.327 (0.265–0.643)	−0.973	0.331	0.011
br	0.686 ± 0.323 (0.499–0.872)	0.408 ± 0.344 (0.215–0.601)	−1.853	0.064	0.538
da	0.310 ± 0.317 (0.128–0.493)	0.442 ± 0.279 (0.281–0.603)	−0.910	0.363	−0.257
dm	0.240 ± 0.266 (0.086–0.393)	0.399 ± 0.266 (0.245–0.553)	−1.569	0.177	−0.359
dp	0.299 ± 0.315 (0.117–0.481)	0.403 ± 0.408 (0.168–0.639)	−0.534	0.594	−0.124
tlh	0.276 ± 0.329 (0.085–0.466)	0.335 ± 0.347 (0.135–0.535)	−0.596	0.511	0.048
tr	0.246 ± 0.214 (0.122–0.370)	0.425 ± 0.329 (0.235–0.616)	**−2.040**	**0.041**	**−0.143**

Note: Anterior Deltoid (da), Medial Deltoid (dm), Posterior Deltoid (dp), Biceps Brachii (bb), Long Head of Triceps Brachii (tlh), Brachioradialis (br), Trapezius Descendens (tr), M = mean, SD = standard deviation, 95% CI = 95% confidence interval, Z = Wilcoxon signed-rank test.

### Performance

The overall task duration did not differ significantly between conditions, with an average of 2.18 ± 0.25 s in the *noExo* condition and 2.24 ± 0.24 s in the *Exo* condition (F = 1.231, *p* =.287, *η²* = 0.086). However, the aiming phase lasted significantly longer when using the exoskeleton (1.46 ± 0.15 s) compared to the *noExo* condition (1.40 ± 0.13 s), with a moderate effect size (*F* = 2.504, *p* =.0138, *η²* = 0.161). In contrast, the duration of the drilling phase remained nearly identical between conditions (0.78 ± 0.18 s vs. 0.782 ± 0.22 s; *F* = 0.001, *p* =.974, *η²* = 0.000).

Regarding accuracy, a notable difference was found in the mean error integral during aiming, which was significantly higher in the *Exo* condition (0.2 ± 0.0) compared to *noExo* (0.1 ± 0.0) (*F* = 73.393, *p* <.001; *η²* = 0.850). This indicates a strong impact of the exoskeleton on aiming precision. In contrast, the mean error integral during drilling showed no significant difference between conditions (*F* = 0.636, *p* =.439, *η²* = 0.047) (cf. Table 2).

**Table 2:** Performance data.

Performance	noExo [M ± SD] (95% CI)	Exo [M ± SD] (95% CI)	Differences [F, *p*, η²]	Effect size (cohen’s d)
task duration (sec)	2.181 ± 0.252 (2.030–2.331)	2.236 ± 0.237 (2.094–2.378)	1.231, 0.287, 0.086	−0.218
duration aiming (sec)	1.403 ± 0.131 (1.324–1.482)	1.460 ± 0.148 (1.371–1.549)	2.504, 0.138, 0.161	−0.390
duration drilling (sec)	0.777 ± 0.180 (0.670–0.886)	0.776 ± 0.223 (0.643–0.909)	0.001, 0.974, 0.000	0.007
error integral aiming	0.083 ± 0.017 (0.073–0.093)	0.221 ± 0.059 (0.187–0.255)	**73.393**,** < 0.001**,** 0.850**	**−3.159**
error integral drilling	0.093 ± 0.042 (0.069–0.118)	0.103 ± 0.054 (0.072–0.134)	0.636, 0.439, 0.047	−0.197

Note: M = mean, SD = standard deviation, F = repeated measures ANOVA, 95% CI = 95% confidence interval.

### RPE and BPDS

**Fig. 3:**
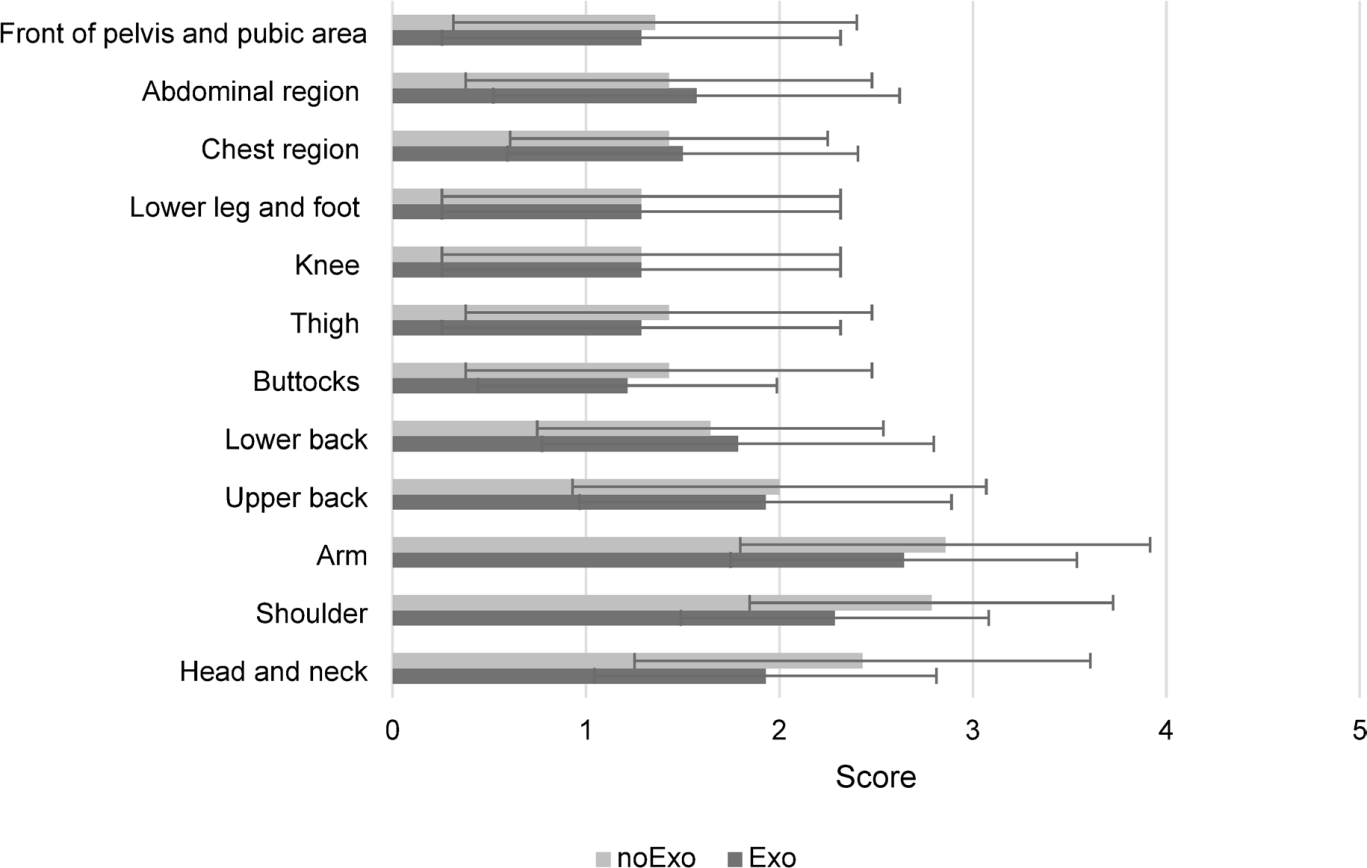
RPE and BPDS results.

Figure 3 presents the results of RPE on the Borg 100-point scale and the BPDS, comparing two conditions: *noExo* (without an exoskeleton) and *Exo* (with an exoskeleton). The overall RPE was slightly lower in the *Exo* condition (32 ± 13) compared to *noExo* (37 ± 16), but this difference was not statistically significant (*Z* = −1.384, *p* =.166). Similarly, most body regions showed no significant differences in BPDS between conditions. However, a notable exception was found for the shoulders, where discomfort was significantly lower in the *Exo* condition (*Z* = −2.111, *p* =.035).

### SUS

The results of the SUS for the evaluation of the exoskeleton showed total scores ranging from 7.5 to 67.5 (28.4 ± 14.8), indicating usability levels from unacceptable to marginally acceptable.

The results indicate that users found the system challenging to learn, as the statement *‘I needed to learn a lot of things before I could get going with this system’* received a high level of agreement. Similarly, many users found the system cumbersome and unnecessarily complex, suggesting difficulties in navigation and ease of use. Confidence in using the system was relatively low, as indicated by the lower scores for *‘I felt very confident using the system’* and *‘I thought the system was easy to use’.* Additionally, users did not strongly agree that they would like to use the system frequently, implying dissatisfaction with its usability. While the integration of various functions received a neutral rating, the responses suggest inconsistencies within the system, as indicated by the agreement with *‘I thought there was too much inconsistency in this system’* (cf. Figure 3).

**Fig. 4:**
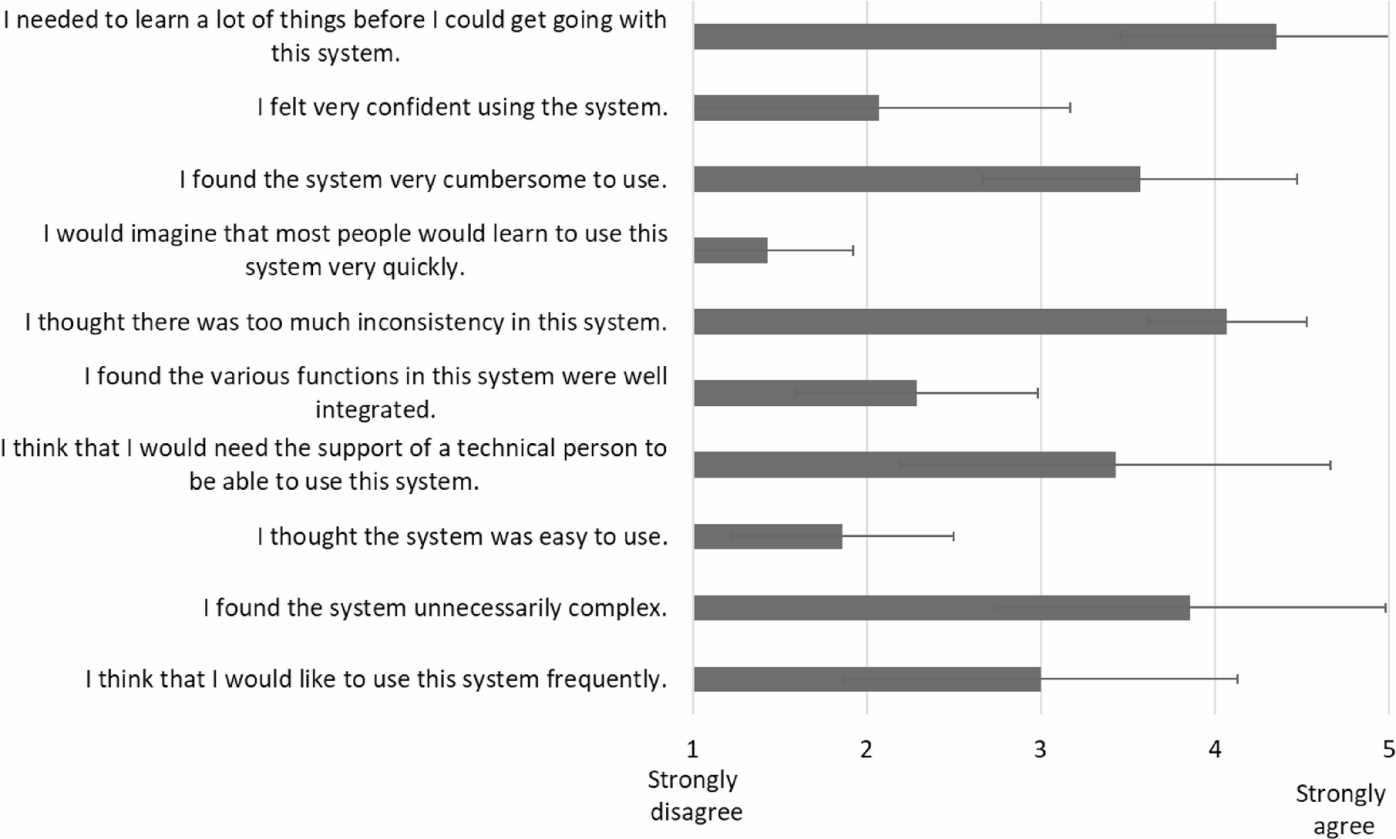
SUS results for Exoskeleton use.

Figure 4 shows the SUS results as bar chart with mean ratings and error bars across ten SUS items. Responses range from strongly disagree (1) to strongly agree (5). Participants reported high agreement with needing to learn a lot before use, finding the system cumbersome, inconsistent, and unnecessarily complex. They disagreed with statements that the system was easy to use, quick to learn, or well-integrated. Confidence in using the system and willingness to use it frequently scored low to moderate.

## Discussion

Physically demanding tasks involving the upper limbs continue to challenge workplace ergonomics and promote interest in assistive technologies, such as exoskeletons. Given that such tasks increase the risk of arm pain by 18%^7^, exoskeletons have been explored as potential solutions for reducing muscular strain and improving work ergonomics^8,12,14,15^, while effects on muscle synergy patterns, task performance and usability remain debated^18^. The present study offers new insights by evaluating these aspects in female participants, revealing a complex interplay between muscle relief, movement constraints, and user adaptation.

A particular strength of this study is its exclusive focus on female users, an underrepresented population in exoskeleton research^11,18^. The observed effects cannot be interpreted as sex-specific due to the absence of a male control group and may also reflect anthropometric and strength-related differences. Comparisons with previous studies remain limited due to differences in equipment, tasks, and methods, as existing studies rely on different experimental designs, activities, and exoskeleton systems, whereas our approach, for example, incorporates weight-adjusted muscle activation based on synergies, which makes direct comparability with previously published results difficult. Importantly, these findings expand the current body of research on exoskeletons by providing gender-specific insights into neuromuscular adaptations, trade-offs in performance, and user-friendliness, thereby underscoring the need to incorporate gender-specific characteristics into both experimental design and device development.

Consistent with previous findings (Gillette and Stephensond, 2018^14,16^, the exoskeleton reduced M. Trapezius activity during arm descent (cluster 3), confirming reduced scapular stabilization demands^42,43^. Effects were strongest in stabilization-related phases (clusters 2 and 3), while no differences were observed during arm lifting (cluster 1).

This phase-specific effect can be interpreted in the context of recent research on muscle synergies. Cohic et al., (2025) demonstrated that support from an exoskeleton does not significantly alter the number or spatial structure of muscle synergies during lifting but rather modulates their activation patterns. This supports the interpretation that the effects observed in our study reflect a rebalancing of existing motor modules rather than a reorganization of the underlying control architecture, which is consistent with the unchanged behaviour in Cluster 1. However, this stability does not preclude task-dependent adaptations. Park and Nussbaum (2025) demonstrated that more complex or mechanically demanding overhead tasks can lead to an increase in the number of recruited synergies, suggesting that the central nervous system flexibly adapts coordination strategies depending on task constraints. This provides a plausible explanation for the enhanced redistribution effects observed in our stabilization-related clusters (2 and 3), where external support likely altered the demands on intermuscular coordination. Tian et al., (2025) have linked these perspectives and shown that while primary synergies remain very stable under all conditions, secondary synergies are more flexible and can be modulated or reactivated. Importantly, they also observed lower muscle activation and more efficient activation patterns under exoskeleton conditions, suggesting that the support primarily enhances neuromuscular efficiency rather than simplifying motor control. This interpretation is further supported by Penna et al.^30^, who demonstrated that muscle synergies are not only stable control units but also contain sufficient information to decode movement intentions in real time.

In line with these findings, our results suggest that the exoskeleton did not uniformly reduce muscle activity but rather may indicate a redistribution of muscular effort across stabilization phases. In cluster 2, the balanced activation across muscles may suggest a more distributed stabilizing demand, although this interpretation is not directly supported by statistical comparisons of intermuscular coordination nor formal statistical test of balance. This may reflect compensatory strategies, as the exoskeleton’s constraints could alter natural joint dynamics^31^. However, these interpretations remain speculative, as neither kinematic data nor direct measures of coordination were collected. The relatively similar mean activations across all muscles support this interpretation and suggest a distributed stabilization strategy. This balanced pattern might also be the result of individual differences in dominant muscle activation strategies, which, when averaged, appear more uniform. Anthropometric and strength differences in female users may also have influenced muscle activation patterns^27,32^. A kinematic analysis of arm trajectories would help clarify how these mechanical constraints influenced motor control and precision.

Despite muscular benefits, the exoskeleton did not improve task efficiency. Contrary to expectations, aiming duration increased, and aiming precision significantly declined. This aligns with Fournier et al.^18^, who found that exoskeleton effects on productivity vary depending on task demands. These effects may result from constrained joint dynamics requiring adapted coordination strategies.

Despite reduced muscle activity, task performance decreased, with longer aiming times and reduced accuracy. This aligns with Fournier et al.^18^, suggesting that exoskeleton effects depend on task demands. Mechanical constraints likely altered joint dynamics and required adapted coordination strategies^31^. Such constraints can impair fine motor control and increase cognitive load^11,44^, potentially affecting accuracy and muscle activity. Early-stage learning effects may also have contributed to the observed performance reduction.

Against this background, usability ratings provide a key explanation for the observed trade-offs between muscle relief and task performance. The low SUS score indicates usability difficulties. This may be linked to suboptimal anthropometric fit, particularly in female users, although findings on sex differences remain mixed^33,34^. This discomfort likely explains why perceived exertion did not decrease despite reduced muscle activity, as poor usability can offset biomechanical benefits^45^. In this context, SUS and discomfort can be interpreted as complementary subjective measures that are sensitive to fit-related limitations and thus help explain the discrepancy between objective biomechanical effects and perceived exertion. Participants might have compensated for mechanical constraints by increasing stabilizing effort, thereby counteracting the expected fatigue relief. However, the available data do not directly indicate an increase in the effort required for stabilization, which is why these results should be interpreted with caution. More generally, comfort, ease of use, and ergonomic fit are key determinants of exoskeleton acceptance^46^. In the present study, low usability and discomfort suggest that ergonomic limitations influenced subjective experience. Even though the fit was not directly quantified, the combination of SUS and discomfort provides indirect but consistent evidence that ergonomic limitations influenced the subjective experience. These limitations may have counteracted the expected benefits in perceived exertion. Given the rising incidence of WMSDs^2,6^, it is crucial to optimize exoskeletons for both ergonomic support and functional usability. Future research should focus on task-specific adaptations, sex-inclusive designs, and long-term adaptation effects. Overall, these findings underscore that women-centred analyses are essential for a comprehensive understanding of the effects of exoskeletons, as they reveal specific interactions between biomechanical unloading, motor control, and user-friendliness that might be overlooked in more heterogeneous or male-dominated samples^47–52^.

While this study offers valuable insights into responses observed in female participants for passive upper-limb exoskeleton use, several limitations should be considered. Limited prior experience and a short familiarization period may have influenced performance and usability outcomes, suggesting that adaptation effects cannot be excluded. Future studies should include longer training protocols. The use of a single passive exoskeleton with fixed assistance limits generalizability to other devices and system. The sample size was below the a priori power analysis by eight participants, resulting in reduced statistical power (post hoc power = 0.26) and limited generalizability.

These limitations have a direct impact on the interpretation of the present results. In particular, results on muscle synergies should be interpreted with caution due to limited statistical power. The lack of kinematic data limits interpretation of underlying movement strategies.

The findings highlight the importance of user-specific design considerations, including strength, anthropometry, and movement patterns. Although shoulder muscle activity was reduced, this was accompanied by decreased accuracy and longer task duration, likely due to mechanical and usability constraints. This underscores the complex relationship between biomechanical relief, motor control, and user experience during exoskeleton-assisted tasks.

Future research should examine sex-specific differences and develop more user-adapted exoskeleton designs, using comprehensive evaluation approaches to balance biomechanical relief, precision, and usability in real-world settings.

## Data Availability

The datasets generated and/or analysed during the current study are available from the corresponding author on reasonable request.

## Appendix

Appendix Table 1. Perceived exertion and dicsomfort.

**Table:**

RPE overall (0–100)	noExo [M ± SD] (95% CI)	Exo [M ± SD] (95% CI)	Differences[Z, *p*]	Effect size (Rank-Biserial Correlation)
	37 ± 16 (28–46)	32 ± 13 (25–40)	−1.384, 0.166	−0.449
Head and neck	2 ± 1 (2–3)	2 ± 1 (1–3)	−1.823, 0.068	−0.810
Shoulders	3 ± 1 (2–3)	3 ± 1 (2–3)	**−2.111**,** 0.035**	**−0.778**
Arms	3 ± 1 (2–4)	3 ± 1 (2–3)	−1.155, 0.248	−0.400
Upper back	2 ± 1 (2–3)	2 ± 1 (1–3)	− 0.378, 0.705	−0.200
Lower back	2 ± 1 (1–2)	2 ± 1 (1–2)	−1.000, 0.317	0.500
Buttocks	1 ± 1 (1–2)	1 ± 1 (1–2)	−1.732, 0.083	−1.000
Tights	1 ± 1 (1–2)	1 ± 1 (1–2)	−1.414, 0.157	−1.000
Knees	1 ± 1 (1–2)	1 ± 1 (1–2)	0.000, 1.000	-
Lower legs and feet	1 ± 1 (1–2)	1 ± 1 (1–2)	0.000, 1.000	-
Chest region	1 ± 1 (1–2)	2 ± 1 (1–2)	0.000, 1.000	0.000
Abdominal region	1 ± 1 (1–2)	2 ± 1 (1–2)	− 0.378, 0.705	0.200
Front of pelvis	1 ± 1 (1–2)	1 ± 1 (1–2)	−1.000, 0.317	−1.000

Note: M = mean, SD = standard deviation, Z = Wilcoxon signed-rank test, 95% CI = 95% confidence interval.
